# From Uncertainty to Clarity: Behçet’s Disease Diagnosed in the Emergency Department

**DOI:** 10.7759/cureus.108158

**Published:** 2026-05-03

**Authors:** Marie Rousseau, Hiba El banouti, Fabien Guérisse

**Affiliations:** 1 Emergency Department, Centre Hospitalier Universitaire (CHU) Charleroi-Chimay, Charleroi, BEL

**Keywords:** aphthous ulcers, arthralgia, behçet’s disease, erythema nodosum, genetic predisposition, uveitis

## Abstract

Behçet’s disease is a complex, multisystem inflammatory vasculitis of unknown etiology, characterized by a highly heterogeneous clinical presentation that may delay recognition, particularly in the emergency setting.

We report a case of a 15-year-old girl presenting to the emergency department with a first episode of painful genital ulceration associated with erythema nodosum and persistent fever. Laboratory investigations revealed a marked inflammatory syndrome without an identifiable infectious cause. Based on clinical findings and according to the International Criteria for Behçet’s Disease (ICBD), a score of 5 was obtained, supporting the diagnosis.

This case underscores the diagnostic challenges associated with early-stage Behçet’s disease, especially in young patients presenting with incomplete or atypical clinical features. It highlights the importance of maintaining a high index of suspicion in the emergency setting to facilitate timely diagnosis and appropriate management.

## Introduction

Behçet’s disease is a rare systemic vasculitis of incompletely understood etiology, characterized by recurrent aphthous ulcerations associated with cutaneous, ocular, articular, neurological, and gastrointestinal involvement. Its clinical presentation is notably heterogeneous and influenced by both age and geographic distribution. In adolescents, early manifestations are frequently limited to mucocutaneous lesions, which are often nonspecific [1,2]. This limited presentation may obscure the diagnosis and contribute to delays.

This clinical case illustrates the diagnostic complexity of Behçet’s disease in its early stages and emphasizes the importance of considering this condition in adolescents presenting with mucocutaneous lesions and systemic inflammatory features in the emergency department.

## Case presentation

A previously healthy 15-year-old girl presented to the emergency department on November 1st with a 10-day history of fever, genital ulcerations, and erythema nodosum. This was her first episode of such symptoms.

She was of Italian origin (Apulia region) on both parental sides. There was no reported family history of autoimmune disease, vasculitis, or Behçet’s disease.

Regarding immunization history, she received a combined injection of Boostrix (diphtheria, tetanus, pertussis) and Trumenba (meningococcal B vaccine) in the right deltoid on October 23, 2025. The following day, she developed localized arm pain, ipsilateral cervical lymphadenopathy, arthralgia, and fever. A cervical ultrasound performed on October 27 revealed right submandibular adenitis. A similar reaction had occurred following a previous Trumenba injection in 2024 and had been interpreted as a local post-vaccination reaction, resolving with symptomatic treatment.

On day six post vaccination, the patient developed a painful vulvar ulcer associated with a probable aphthous lesion. She denied any sexual activity. This was accompanied by headache, myalgia, persistent fever (38-39°C), and the progressive onset of erythema nodosum affecting the lower limbs.

She remained in good general condition. Cervical lymphadenopathy and oral aphthosis resolved with symptomatic treatment (nonsteroidal anti-inflammatory drugs and paracetamol), although the erythema nodosum worsened.

She presented to the emergency department on four occasions over a 10-day period due to persistent symptoms. An initial diagnosis of bartholinitis was made, and amoxicillin-clavulanic acid was prescribed, of which only one dose was taken. A gynecological evaluation on October 30 included a vaginal swab and a nasopharyngeal sample, both of which were negative.

Laboratory investigations demonstrated a marked inflammatory syndrome, characterized by a progressive elevation of C-reactive protein levels in the absence of significant leukocytosis (Table 1), thereby suggesting a non-infectious etiology. This discrepancy between pronounced biological inflammation and the lack of an identifiable infectious source contributed to broadening the differential diagnosis.

**Table 1:** Laboratory results.

Test	Results		Reference range
	October 28	November 1	
Hemoglobin	12.9 g/dL	12.2 g/dL	12.0-15.0
White blood cells	5500/µL	4670/µL	4000-10000
Neutrophils	4060/µL	3350/µL	2000-7000
Lymphocytes	820/µL	1090/µL	1000-3000
C-reactive protein (CRP)	39.6 mg/L	67.5 mg/L	<5.0

No additional laboratory or serological results were available at the time of admission. There were no objective signs of inflammatory arthritis or ocular, gastrointestinal, or neurological involvement.

According to the International Criteria for Behçet’s Disease (ICBD) [3], the patient had a score of 5, based on the presence of oral aphthosis and, at presentation, genital ulceration and erythema nodosum [4].

## Discussion

Pathophysiology

Behçet’s disease is a multisystem autoinflammatory vasculitis characterized by involvement of vessels of all sizes. It predominantly affects young adults, particularly males, in Mediterranean and Middle Eastern regions [1,2,5,6].

The pathogenesis involves a complex interplay between genetic susceptibility and environmental triggers. Certain individuals, particularly those carrying the HLA-B51 allele or polymorphisms in genes such as IL10, IL23R, and ERAP1, exhibit impaired regulation of inflammation and immune tolerance [1,2,5].

Environmental triggers, including infections with *Streptococcus* species or herpes simplex virus type 1, may induce an exaggerated immune response through molecular mimicry, in which microbial antigens are mistakenly recognized as host structures [1].

These mechanisms lead to endothelial dysfunction and vascular inflammation. Endothelial cell apoptosis exposes subendothelial structures, promoting platelet activation and thrombus formation. Chronic inflammation further weakens arterial walls, predisposing to aneurysm formation [1,5,7].

Diagnosis and investigations

The diagnosis of Behçet’s disease remains primarily clinical [2]. The International Criteria for Behçet’s Disease (ICBD) are currently the most widely used diagnostic tool, demonstrating high sensitivity and specificity; a score ≥4 is considered strongly suggestive of the disease (Table 2) [3,4,6,8].

**Table 2:** International classification criteria for Behcet's diagnosis. * Pathergy test is optional.

Symptom	Points
Ocular lesions	2
Oral aphthosis	2
Genital aphthosis	2
Skin lesions	1
Neurological manifestations	1
Vascular manifestations	1
Positive pathergy test *	1

To date, no pathognomonic test exists. Investigations are therefore mainly aimed at supporting the diagnosis and excluding alternative diagnoses [2].

Laboratory findings may include inflammatory anemia and elevated CRP levels, particularly during active disease. The presence of HLA-B51 may indicate increased susceptibility but lacks specificity.

The pathergy test assesses cutaneous hyperreactivity and involves performing two to three superficial needle pricks (typically on the forearm) using a sterile needle (without product) [2,6,8,9]. A positive test is defined by the development of an erythematous papule or pustule (2-5 mm) within 24-48 hours, reflecting exaggerated neutrophilic activation. However, its sensitivity varies geographically and is generally low in Western Europe, limiting its diagnostic utility [6,8].

Clinical findings should guide additional investigations and may include ophthalmologic evaluation (fundoscopy, optical coherence tomography), venous Doppler ultrasound (for thrombosis), CT angiography (for arterial aneurysms), brain magnetic resonance imaging (for neurological involvement), colonoscopy with biopsies, and abdominal imaging (for gastrointestinal involvement) [2].

Diagnosis is often delayed due to nonspecific early manifestations, including recurrent oral and genital ulcers, erythema nodosum, uveitis (sometimes with hypopyon), arthralgia, and diverse cutaneous lesions [2,6].

Differential diagnosis

The differential diagnosis of Behçet’s disease is broad and depends on the type of clinical manifestation.

In the presence of mucosal involvement, particularly oral and genital ulcerations, several conditions should be considered, including inflammatory bowel diseases such as Crohn’s disease and ulcerative colitis, celiac disease, viral or bacterial infections (notably herpes simplex virus, syphilis, and HIV), autoimmune disorders such as systemic lupus erythematosus and pemphigus vulgaris, as well as nutritional deficiencies involving vitamin B12, folate, iron, or zinc. Chronic neutropenia and drug-induced ulcerations, particularly related to medications such as methotrexate or niflumic acid, should also be considered [9,10].

When ocular involvement is present, including uveitis, hypopyon, or retinitis, the main differential diagnoses include ankylosing spondylitis, sarcoidosis, systemic lupus erythematosus, ocular infections such as syphilis or tuberculosis, and multiple sclerosis, particularly in cases associated with optic neuritis or central nervous system involvement [9,11].

Cutaneous manifestations such as erythema nodosum or pseudofolliculitis should prompt consideration of other conditions, including pyoderma gangrenosum, acne fulminans, idiopathic or infection-related erythema nodosum, sarcoidosis, and inflammatory bowel disease [12,13].

In the presence of articular symptoms such as arthralgia, the differential diagnosis includes seronegative spondyloarthropathies, rheumatoid arthritis, systemic lupus erythematosus, and reactive (post-infectious) arthritis [9,14].

Vascular involvement, including thrombosis or aneurysm formation, may be observed in Behçet’s disease but should also raise suspicion for antiphospholipid syndrome, other systemic vasculitides such as polyarteritis nodosa or antineutrophil cytoplasmic antibody (ANCA)-associated vasculitis, inherited thrombophilia, as well as infectious causes, including mycotic or tuberculous aneurysms [9,13].

Finally, in cases of neurological involvement, Behçet’s disease must be distinguished from multiple sclerosis, neurosarcoidosis, infectious meningitis, isolated cerebral venous thrombosis, and secondary cerebral vasculitis, particularly in the context of systemic lupus erythematosus [9].

Treatment

The type and severity of organ involvement primarily guide the management of Behçet’s disease. In patients with predominantly mucocutaneous and articular manifestations, colchicine remains the cornerstone of first-line therapy, as it has been shown to reduce the frequency and severity of disease exacerbations. In cases of intolerance or contraindication to colchicine, treatment is mainly symptomatic, with nonsteroidal anti-inflammatory drugs providing relief of pain and inflammation, although they do not modify the underlying disease course [2].

For patients with more severe or refractory disease, particularly in the presence of ocular, neurological, or vascular involvement, escalation to systemic corticosteroids and immunosuppressive agents such as azathioprine, cyclosporine, or cyclophosphamide may be required. In recent years, biologic therapies, particularly anti-tumor necrosis factor agents, have demonstrated efficacy in refractory forms and are increasingly used in severe disease [2].

In our case, management was limited to nonsteroidal anti-inflammatory drugs due to intolerance to colchicine, with a favorable initial clinical response.

## Conclusions

Establishing a diagnosis of Behçet’s disease within the emergency department remains challenging. Its polymorphic presentation and the absence of a specific diagnostic test require a thorough and detailed clinical assessment to avoid misdiagnosis. Diagnosis is primarily based on clinical findings, supported by the ICBD criteria, which offer good sensitivity and specificity, with a score ≥4 being highly suggestive. In the absence of a definitive diagnostic test, investigations are mainly directed toward excluding alternative diagnoses.

First-line therapy for Behçet’s disease with predominantly mucocutaneous and articular involvement is based on colchicine, which has been shown to reduce the frequency and severity of disease flares. In our case, the patient was managed with nonsteroidal anti-inflammatory drugs for symptomatic relief.
